# Comparison between Fissure Sealant and Fluoride Varnish on Caries Prevention for First Permanent Molars: a Systematic Review and Meta-analysis

**DOI:** 10.1038/s41598-020-59564-5

**Published:** 2020-02-13

**Authors:** Feifei Li, Peipei Jiang, Fanyuan Yu, Chunjie Li, Si Wu, Jing Zou, Xin Xu, Ling Ye, Xuedong Zhou, Liwei Zheng

**Affiliations:** 1 State Key Laboratory of Oral Diseases, National Clinical Research Center for Oral Diseases, Department of Pediatric Dentistry, West China Hospital of Stomatology, Sichuan University, Chengdu, China; 2 West China School of Stomatology, Sichuan University, Chengdu, China; 3 National Key Clinical Specialty on Endodontics, West China Hospital of Stomatology, Sichuan University, Chengdu, China

**Keywords:** Dental caries, Fluoridation, Preventive medicine

## Abstract

The high prevalence and heavy socio-economic burden for caries of first permanent molars (FPMs) make the prevention of this disease a major public health goal. Current guidelines recommend a preference of fissure sealant (FS) over fluoride varnish (FV) based on two recent systematic reviews. However, evidences of these two studies are weak because of scarce data and some limitations. Besides, an up-to-date large scale randomized controlled trial (RCT) reported commensurate effectiveness of these two techniques. Thus, in order to more accurately compare the clinical efficacy between FS and FV on caries prevention for FPMs, we carried out this systematic review and meta-analysis. A total of 8 RCTs involving 3289 participants and 6878 FPMs fulfilled the inclusion criteria. Our meta-analysis for the first time showed that there was no statistical difference on caries incidence or occlusal DMFS increment between sealant group and fluoride varnish group at 2~3 years’ follow-up. In that sense, biannual applications of FV or FS may be equally effective on caries prevention for FPMs. These results do not support routine recommendation of FS over FV, thus shedding light on current conceptions. Our findings endow clinicians with a window to reconsider the choice between these two techniques.

## Introduction

Caries is one of the most prevalent chronic diseases worldwide^1–5^. Approximately 2.4 billion people, accounting for 35% of the world population, are affected by untreated caries^1,2^. Though enormous public resources have been poured into caries prevention, the global prevalence of untreated caries remained stagnant during the last decades^1^. Untreated caries can be much burdensome socio-economically^3–5^. The economic loss thereof amounts to hundreds of billions of dollars every year^3^. As a matter of fact, the high prevalence associated with heavy disease burden make caries prevention a major public health goal.

First permanent molars (FPMs) are of great significance to caries prevention^6,7^. Decay of FPMs constitutes the biggest component of decayed missing filled tooth index (DMFT) among children and adolescents^8,9^. As well as being highly prevalent, caries of FPMs also imposes an overwhelming disease burden. FPMs are the keys to establish permanent occlusion^10^. Severe FPMs caries often cause pain and infection, diminished dietary intake and malocclusion^1,8–10^.

At present, two techniques, namely fluoride varnish (FV) and fissure sealant (FS), are introduced to prevent caries of FPMs, both proved effective^11,12^. However, it still cannot be concluded clearly which of these two skills is clinically superior. Current practice guidelines recommend a preference for FS over FV^6,7^. This recommendation was supported by two very recent systematic reviews. Meta-analysis of them favored FS rather than FV with statistical significance^6,7^. But the evidences of these two studies were assessed as weak (Details of their limitations will be identified and discussed below). Besides, a large-scale, high-qualified RCT updated recently showed that there was no significant difference on prevention efficacy of FPMs caries between FV and FS^13^. Such weakened validity of previous systematic reviews as well as new opposing evidences of RCT prompted us to review the reliability of previous studies, providing a strong rationale to conduct present meta-analysis. In summary, our study aimed to accurately evaluate the efficacy of FPMs caries management between FS and FV.

## Results

### Search results

**Figure 1:**
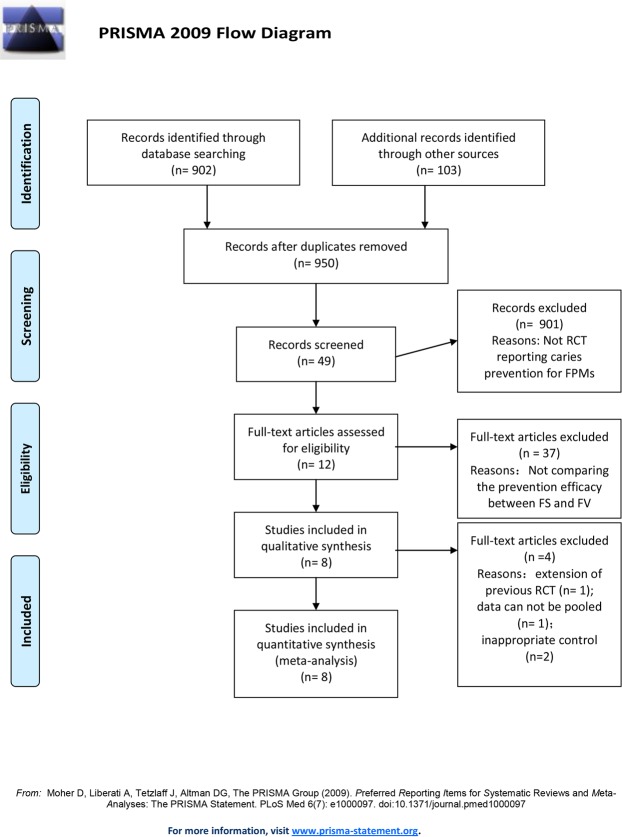
Search results and flow-chart of study selection for quantitative syntheses.

A total of 950 studies surfaced during the search procedures. After selection according to the inclusion and exclusion criteria, 8 studies were included for quantitative synthesis (meta-analysis), yet five of these 8 studies were not included in previous meta-analysis (Table 1)^13–20^. RCTs included in present study dated from 1984 to 2017, which enrolled in 3289 participants and 6878 FPMs. The details of search procedure were presented in study flowchart (Fig. 1). Characteristics of study design, basal conditions of enrolled participants, follow-up period, intervention, and controls of included RCTs were demonstrated in detail in Table 1. Summary of studies excluded in quantitative synthesis and the reasons of exclusion were provided in Table 2.

**Table 1:** Characteristics of included and excluded studies. –: no information available; F/M: female number versus male number; F/U, Follow-up; FS: fissure sealant; FV: fluoride varnish; FPMs: first permanent molars; CI: caries incidence; DMFS: decayed, missing, filled tooth surface; ICDAS: International Caries Detection and Assessment System; GIC: glass ionomer cement; RMGIC: resin modified glass ionomer cement; N/A: no application; OHE: oral health education.

STUDY ID	STUDY TYPE (STUDY DESIGN)	PATIENT				ARMS			REPLACEMENT of SEALANT	F/U PERIOD (MONTHS)	POOLED OUTCOMES
		NUMBER	AGE (YEAR)	GENDER (F/M)	ICDAS CODE	FS	FV	CONTROL			
**RCTs both included in our quantitative synthesis and that of previous two systematic reviews**											
Liu 2012	RCT, parallel	501	9.1	250/251	0–2	121 resin-based	116 22,600ppm	124 Water	N/A	24mons	CI
Bravo 1996	RCT, parallel	362	6–8′	177/185	0	75 resin-based	77 22,600ppm	94 Blank	YES	24mons	CI
Raadal 1984	RCT, split-mouth desgin	121	6–9′	62/59	0–3	210 resin-based	210 22,600ppm	N/A	N/A	23mons	CI
**RCTs updated in our quantitative synthesis**											
Chestnutt 2017	RCT, parallel	1015	6–7′	543/472	0	514 resin-based	501 22,600ppm	N/A	YES	36mons	CI
Salem, 2014	RCT, parallel	400	6–7′	—	0	200 resin-based	200 22,600ppm	N/A	YES	24mons	CI DMFS increment
Bravo 1997	RCT, parallel	362	6–8′	177/185	0	100 resin-based	98 22,600ppm	116 Blank	YES	24mons	DMFS increment
Tagliaferro 2011	RCT, parallel	268	6–8′	129/139	0	91 RMGIC	91 22,600ppm	86 OHE	N/A	24mons	DMFS increment
Ji 2007	RCT, parallel	622	6–8′	—	0	205 GIC	207 7,700ppm	210 Blank	N/A	36mons	CI
**RCTs excluded in our quantitative synthesis but included in previous two systematic reviews**											
Bravo 2005	RCT, parallel	362	6–8′	177/185	0	37 resin-based	38 22,600ppm	45 Blank	YES	108mons	CI
Florio 2001	RCT, parallel	34	6	—	1–3	12 RMGIC	11 22,600ppm	11 OHE	N/A	12mons	caries progression% caries arrestment%
Houpt 1983	RCT, split-mouth desgin	205	6–10′	—	0	313 resin-based	N/A	313 Blank	N/A	72mons	CI

**Table 2:** Summary of studies excluded in quantitative synthesis and the reasons of exclusion.

Excluded Study ID	Reasons of exclusion
Bravo 2005	This study reported the results at the endpoint of 9th year. However biannual application of fluoride varnish in FV group was terminated early from 4th year.
Florio 2001	The data provided in this study cannot be pooled and quantitatively synthesized (No data on caries incidence or occlusal DMFS increment were provided) (Table 1).
Houpt 1983	No fluoride varnish was applied to control group.
Splieth 2001	The interventions in this study did not meet our inclusion criteria (This study compared caries prevention efficacy between fissure sealant plus fluoride varnish and fluoride varnish alone).

### Assessment of the risk of bias

**Figure 2:**
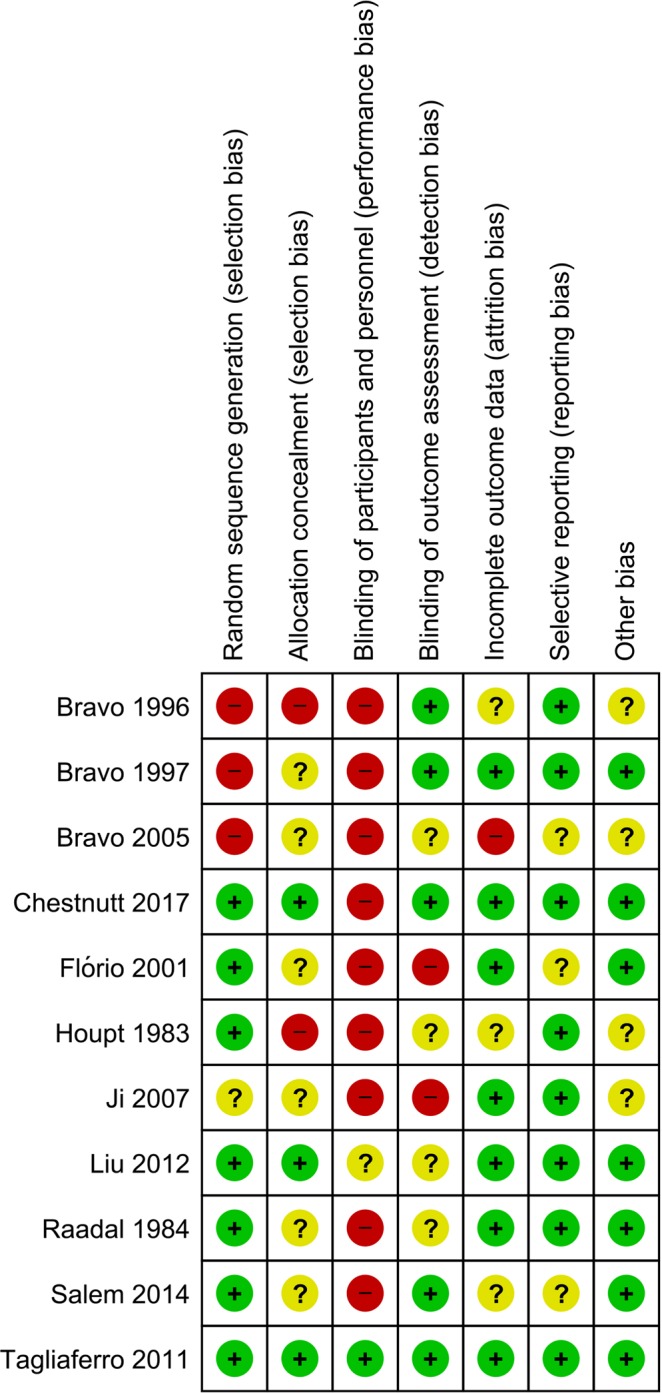
Bias assessment of included studies.

Among included RCTs, only Bravo *et al*. 1996 & 1997 & 2005 exhibited high risks of multiple bias^19–21^. Bravo 2005 was the extended research of Bravo 1997 and Bravo 1996^21^. The dropout ratio of Bravo 2005 added up to more than 60%, and there were 3.9% molars of FV group in this study presented sealant at the end of follow-up. Therefore the reliability of Bravo 2005 was severely impaired^21^. Besides Bravo 2005 reported the results at the endpoint of ninth year, whereas the biannual application of fluoride varnish in FV group was terminated early from the 4th year. For above reasons Bravo 2005 was excluded in present meta-analysis^21^. Results of bias assessment were presented in Fig. 2.

### FS or FV and caries incidence (CI)

CI is the primary outcome to measure caries prevention efficacy. Six of included RCTs reported this outcome^13–16,18,19^. Three layers of CI were demonstrated, including CI of enrolled children, CI of FPMs, and CI of FPMs’ occlusal surfaces. Chestnutt 2017 also reported CI of nonocclusal surfaces of FPMs, but no other RCTs provided this outcome, therefore quantification of this parameter cannot be made in our study^13^.

### CI of enrolled children

**Figure 3:**
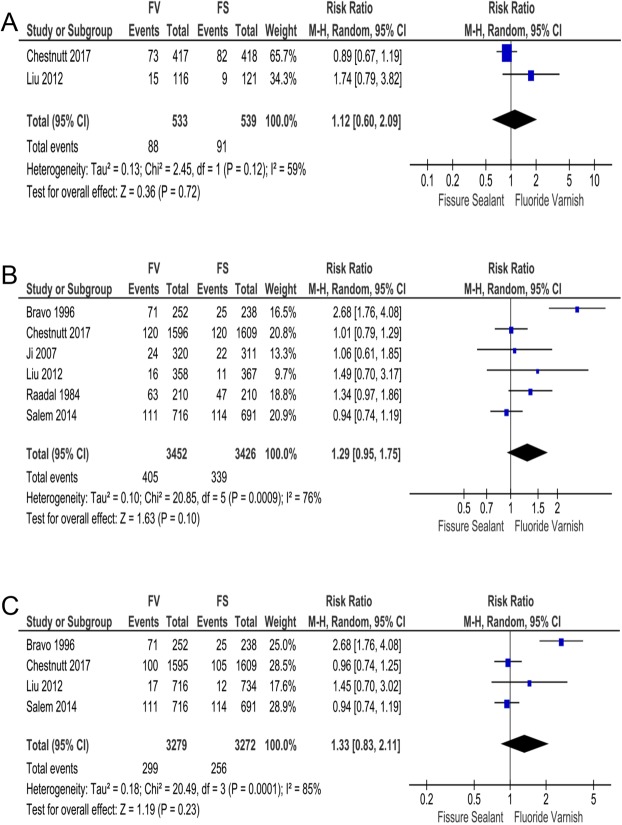
Meta-analysis for caries incidence (CI) after 2~3 years of follow-up. Forest plot of comparison: FS groups was compared with FV groups with respect to (**A**) CI of enrolled children (**B**) CI of FPMs (**C**) CI of FPMs’ occlusal surfaces.

Two RCTs reported this outcome, which included 1072 participants^13,18^. We pooled data from these two RCTs and our results showed the overall relative ratio (RR) of CI for FS to FV was 1.12 without statistical significance (95% CIs: 0.60 to 2.09; *p* = 0.72). Heterogeneity of this meta-analysis was high (x^2^ = 2.45, I^2^ = 59%) (Fig. 3A).

### CI of FPMs

Six included RCTs reported this outcome^13–16,18,19^. These RCTs included a total of 6878 FPMs. Among them only Bravo 1996 reported statistical superiority of FS on reducing CI of FPMs (RR: 2.68, 95% CIs: 1.76 to 4.08)^19^. Others showed no significant difference^13–16,18^. Our overall RR of this outcome lacked statistical significance to show superiority between FV and FS (RR: 1.29; 95% CIs: 0.95 to 1.75; *p* = 0.10). The heterogeneity of this meta-analysis was high (x^2^ = 20.85, I^2^ = 76%) (Fig. 3B).

We also conducted subgroup analysis regardless of Bravo 1996, because of its high bias risk and substantial contribution to heterogeneity of this outcome. Results showed that exclusion of Bravo 1996 did not change overall effect (RR: 1.05; 95% CIs: 0.91 to 1.22, *p* = 0.48), but significantly reduced heterogeneity (x^2^ = 3.92, I^2^ = 0%) (Fig. S2A).

### CI of FPMs’ occlusal surfaces

Occlusal caries are responsible for most decay of FPMs, therefore CI of FPMs’ occlusal surfaces is of great importance in evaluating caries prevention efficacy of FS and FV. Four RCTs reported this outcome^13,16,18,19^. Chestnutt 2017 and Salem 2014 reported that FV exhibited lower occlusal CI than FS without significance^13,16^. In details, RR of Chestnutt 2017 was 0.96 (95% CIs: 0.74 to 1.25), and RR of Salem 2014 was 0.94 (95% CIs: 0.74 to 1.19) (Fig. 3C). Conversely, only Bravo 1996 favored FS with statistical power for it reduced more occlusal caries^19^. RR of this outcome for FV to FS in Bravo 1996 was 2.68 (95% CIs: 1.76 to 4.08) (Fig. 3C). In Liu 2012, this parameter was 1.45 (95% CIs: 0.70 to 3.02) without statistical significance (Fig. 3C). Our overall effect showed that FS only had very limited superiority in reducing occlusal CI compared with FV (RR: 1.33). But it did not reach statistical significance (95% CIs: 0.83 to 2.11, *p* = 0.23). The heterogeneity of this meta-analysis was high (x^2^ = 20.49, I^2^ = 85%) (Fig. 3C).

Because of aforementioned reasons, subgroup analysis with Bravo 1996 excluded was conducted and showed slight superiority of FV in decreasing occlusal CI (RR = 0.98), but no statistic significance was detected (95% CIs: 0.82 to 1.16; *p* = 0.78) (Fig. S2B). Notably, heterogeneity obviously declined after removing Bravo 1996 (x^2^ = 1.24, I^2^ = 0%) (Fig. S2B).

### FS or FV and occlusal DMFS increment of FPMs

**Figure 4:**
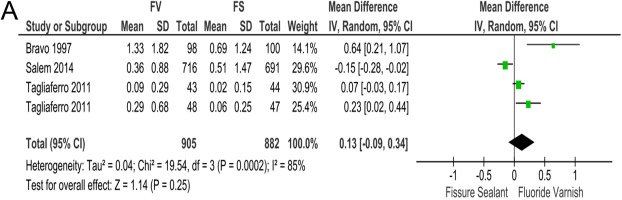
Meta-analysis for occlusal DMFS increment of FPMs after 2~3 years of follow-up. Forest plot of comparison: FS groups was compared with FV groups with respect to occlusal DMFS increment of FPMs.

DMFS increment of occlusal surfaces is the secondary outcome to evaluate caries prevention efficacy of FS and FV. It was reported in three RCTs^16,17,20^. Among these three RCTs, only Bravo 1997 reported higher DMFS increment in FV group with statistical power (MD = 0.64; 95% CIs: 0.21 to 1.07)^20^. However, the rest two RCTs did not achieve statistical significance (Fig. 4). Our meta-analysis did not reach statistical significance to show any superiority between FV and FS in reducing DMFS increment (MD = 0.13; 95% CIs: −0.09 to 0.34; *p* = 0.25). The heterogeneity of this meta-analysis was high (x^2^ = 19.54, I^2^ = 85%) (Fig. 4).

For this outcome, as the huge contribution of Bravo 1997 to heterogeneity, we conducted subgroup analysis excluding this study. Final results showed consistent conclusion as that of analysis including this study, that is, no statistic difference was detected between FV and FS on this parameter (MD = 0.04; 95% CIs: −0.15 to 0.23; *p* = 0.70) (Fig. S2C). Removing Bravo 1997 slightly reduced heterogeneity (x^2^ = 11.90, I^2^ = 83% (Fig. S2C).

### Analysis without split mouth design

Among included RCTs, only Raadal 1984 adopted split mouth design (Table 1)^14^. It should be noted that in split-mouth study designs, sealed teeth may be impacted by elevated fluoride concentration in saliva^6,22^. Meanwhile, teeth in FV group may also benefit from FS induced oral hygiene improvement^23^. Thus, in order to rule out this carry-over effect, we did supplemental meta-analysis that excluded Raadal 1984. Actually removal of split mouth design did not change the overall effect on CI of FPMs. In details, overall RR about CI of FPMs was 1.29, and it was not statistically different (95% CIs: 0.88 to 1.87; *p* = 0.19). The heterogeneity of this analysis was high (x^2^ = 20.01, I^2^ = 80%) (Fig. S1). In summary, whether or not Raadal 1984 was excluded this did not change the assessment of caries prevention efficacy between FV and FS.

## Discussion

Caries is the most prevalent oral disease around the world^1–5^. It poses a huge economic burden and undermines quality of life^1,2^. FPMs, the keys of permanent dentition, are the most susceptible permanent teeth to caries in school children and adolescents^8–10^. Caries prevention of FPMs is thus a massive public health issue^6,7^. At present, both FS and FV have been proved to be effective anti-caries skills^11,12^. However, controversy about their relative efficacy still exists^6,7,13^. This debate may impede public policy on caries prevention. Results of this meta-analysis first of time showed that compared with FS, FV was not significantly associated with higher CI or more DMFS increment in 6–9 years old children at 2–3 years’ follow-up. These findings were contrary to the latest two systematic reviews and current practice guidelines. These results endow clinician with windows to reconsider the choice between these two techniques. FV shows better economic efficacy, requires much lower technique sensitivity and does not need modern dental equipments^16,18,24^. Taking results of present study into consideration, it is recommended that FV could be the more affordable and suitable prevention method for underdeveloped and developing areas.

A meta-analysis by Wright 2016 reported that FS was superior to FV for caries prevention of FPMs^7^. However, this study suffered obvious weaknesses as follows. First, they missed three important RCTs, namely Raadal 1984, Ji 2007, and Salem 2014^14–16^. Each of these studies showed no significant difference between FS and FV, therefore incomplete data rendered conclusion of Wright 2016 inaccurate. Besides, Wright 2016 inappropriately included Houpt 1983, an RCT in which no FV was applied to control teeth^25^. Thus, inappropriate inclusion and exclusion of studies potentially led to excessively exaggerated anti-caries efficacy of FS when compared with FV. Another systematic review from Ahovuo-Saloranta *et al*. also favored FS^6^. Nevertheless, validity of this research was severely damaged by its questionable inclusion of data. Three RCTs, which showed no statistical significance between FS and FV, were excluded in quantitative synthesis of Ahovuo-Saloranta 2016^15–17^. Even though each of these three RCTs met inclusion criteria of Ahovuo-Saloranta 2016 and Tagliaferro 2011 and Salem 2014 were assessed as high quality by this systematic review^6,16,17^. Unreasonable exclusion of RCTs which did not support FS misrepresented overall effect. Another possible reason for diverges in conclusions of earlier meta-analysis and the present meta-analysis was that a recently updated RCT of high quality reported equivalent effectiveness of FS and FV on FPMs caries prevention^13^. This finding may be attributable to lack of statistical significance in our meta-analysis.

Ahovuo-Saloranta 2016 stated substantial heterogeneity between included studies and attributed it to scarce and clinically diverse data available^6^. However, this explanation is untenable. A total of 7 RCTs were included in our meta-analysis. These RCTs together recruited 6878 FPMs in 3289 participants^13–20^. Among them, only Bravo 1996, 1997 & 2005 favored FS with statistical superiority^19,20^. However, these three studies were assessed as low quality and high risk of bias both by previous systematic reviews and us because of their unclear and incomplete randomization procedure and poor allocation concealment. Therefore the reliability of these three studies was severely impaired. Our supplemental meta-analysis showed that exclusion of Bravo 1996 and Bravo 1997 did not alter the overall effect in all outcomes, however dramatically decreased the heterogeneity. Hence it is clear that excluding qualified RCTs which did not show difference between FV and FS but including Bravo 1996 and Bravo 1997 renders previous systematic reviews suffering from huge heterogeneity and inaccurate conclusion. Additionally, we noticed Radaal 1984 obtained split-mouth degsign, but the rest of included RCTs adopted parallel design^14^. Concerning the possible influence of different design, we did supplemental analysis that excluded trial of split-mouth design and showed that exclusion of Radaal 1984 did not alter the overall results.

Compared with previous studies, present meta-analysis is promoted in certain aspects. We did quantitative synthesis using original raw data rather than odds ratio (OR) of caries risk^6,7^. This method permitted more evaluating indices including caries incidence of children, FPMs or occlusal surfaces of FPMs and increment of occlusal DMFS, thus providing more information. It is worthwhile to examine this matter in some detail. Our meta-analysis for the first time showed that there was no significant difference on caries incidence or increment of occlusal DMFS between FS and FV at 2~3 years’ follow-up. The results are contrary to that of the latest two systematic reviews and what many dentists would have expected. But before a solid conclusion could be made, more highly qualified RCTs are still called for, especially those reporting the long-term effect. Though Bravo 2005 provided the data at the endpoint of 9th year, biannual application of fluoride varnish in FV group was terminated early from the 4th year in this study^21^. Thus, the long-term superiority between FS and FV in caries prevention of FPMs is still unproven.

In conclusion, present meta-analysis for the first time proved that biannual application of FV compared with FS results in non-significant difference about caries prevention efficacy of FPMs at 2~3 years’ follow up. These findings do not support routine recommendation of FS over FV, which is new to current conceptions. Future choices between these two skills may rely on technique sensitivity, accessibility and cost of these two treatments in local community.

## Material and Methods

The research strategies used in present meta-analysis were modified from our previous reports^26,27^. All meta-analysis was carried out in accordance with Preferred Reporting Items for Systematic Reviews and Meta-Analyses (PRISMA) and the Cochrane Handbook for Systematic Reviews of Interventions^28,29^.

### Inclusion criteria

Inclusion criteria consisted of PICOS, in details which were participants, interventions/controls, outcome measurements, and study types.

### Types of studies

Only RCTs were included in this systematic review. Other studies, including Quasi-RCTs, controlled clinical trials, cohort studies, case reports were excluded.

### Types of participants

RCTs were included in which the children were 6–9 years and generally healthy. Children in included RCTs should present at least one fully erupted FPM with deep fissures or fissures with signs of early enamel caries, amounting to ICDAS (International Caries Detection and Assessment System) code 0–3^30,31^.

### Types of interventions

In included RCTs interventions of managing FPMs caries must contain FS and FV. In FS group, either resin-based sealant or glass ionomer (GIC) sealant could be utilized. In FV group, fluoride varnish must be used biannually. The procedures of FS or FV should rigorously follow standard protocols of manufacturers.

### Types of outcome measures

In our meta-analysis two independent and parallel outcomes were applied to compare prevention efficacy of FS and FV on FPM caries. The first outcome was caries incidence (CI), including the proportion of children/FPMs/occlusal surfaces of FPMs developing dentin caries (ICDAS 4–6)^13,19,31^. The second outcome was increment of decayed missing filled occlusal surface (DMFS) at the end of follow-up^13,20^.

### Exclusion criteria

Published clinical trials were excluded if they did not meet the above criteria. Besides, RCTs which did not provide data about CI or DMFS were also excluded. Studies excluded in quantitative synthesis in present meta-analysis and the reasons of exclusion were shown in Table 2^9,21,25,32^.

### Search methods

The search was restricted to articles written in English or Chinese. A literature search was carried out within the Cochrane Central Register of Controlled Trials (CENTRAL; 2018), MEDLINE (via OVID, 1948 to February 2018), PUBMED (1960 to February 2018), Embase (1984 to February 2018), and CNKI (2018). The online databases of the Journal of Dental Research, the Journal of Dentistry, the International Journal of Paediatric Dentistry, European Journal of Paediatric Dentistry, and the Community Dentistry and Oral Epidemiology were also searched. Reference lists of relevant articles were checked. In order to find ongoing clinical trials, the World Health Organization International Clinical Trials Registry Platform was searched. The MeSH heading words and free text words were combined. They included “children”, “first permanent molars”, “FPM”, “molar”, “tooth”, “fissure sealant”, “pit and fissure sealant”, “sealant”, “fluoride varnish”, “fluoride” and “varnish”. We combined these words with synonyms for “caries”, “dental caries”, “tooth decay”, and “caries prevention”. Search strategies were finally combined with the Cochrane Highly Sensitive Search Strategy to identify RCTs.

### Study inclusion

Four independent reviewers screened and evaluated the titles and abstracts of all potential articles according to the pre-established selection criteria. Then full-texts were further assessed if a study possibly met the inclusion criteria or it was difficult to make a final decision because of insufficient information. When disagreements came up, they were resolved by consensus, and an alternative investigator acted as an arbiter when no consensus was reached.

### Assessment of bias

The Cochrane “risk of bias” instrument was used to assess the risk of bias. This evaluation was performed by three independent reviewers. Disagreements between estimators were resolved by discussion until consensus was reached. The risk of bias was classified into three categories:

- Low risk of bias if all domains were marked as “low risk”;
- Moderate risk of bias if no domain was marked as “high risk” but at least one were coded as “unclear risk”;
- High risk of bias if one or more domains were marked as “high risk”.

### Data extraction

The following data were extracted: demographic data, method of randomization, randomization concealment and blinding, outcomes (CI, occlusal DMFS increment). Three estimators independently extracted data from the included studies using a custom-designed form.

### Assessment of heterogeneity

Overall, clinical heterogeneity was assessed qualitatively. Patients, design, setting, and intervention characteristics were taken into consideration. Methodological heterogeneity was evaluated via the Risk of Bias tool. Efforts were made, where possible, to estimate reporting biases according to the recommendations from the Cochrane Collaboration tool.

### Statistical analysis

Statistical analysis was carried out utilizing Review Manager 5.1. Heterogeneity was assessed via the I^2^ statistic (a test for heterogeneity) on the level of α = 0.10. If there was considerable or substantial heterogeneity (I^2^ > 50%), a random-effects model was adopted; otherwise a fixed-effects model was used. The results of intervention effect were presented as relative risk (RR) utilizing 95% confidence intervals (CIs). All tests were 2-tailed, and *P* ≤ 0.05 was considered statistically significant.

## Supplementary information

Supplemental information.

## Data Availability

This study is a RCTs-based meta-analysis. All original raw data are provided and accessible in RCTs included in this study (Table 1).
