# Supplementary material for: Comparison between Fissure Sealant and Fluoride Varnish on Caries Prevention for First Permanent Molars: a Systematic Review and Meta-analysis
Source: Sci Rep. 2020 Feb 13;10:2578. doi: 10.1038/s41598-020-59564-5 (PMC7018844; doi:10.1038/s41598-020-59564-5)
Supplement: Supplementary file 1 — Supplemental information. [file 41598_2020_59564_MOESM1_ESM.docx]

**Comparison between Fissure Sealant and Fluoride Varnish on Caries Prevention for First Permanent Molars: a Systematic Review and Meta-analysis**

Feifei Li^1,2^, Peipei Jiang^1,2^, Fanyuan Yu^1,2,3^, Chunjie Li^1,2^, Si Wu^1,2^, Jing Zou^1,2^, Xin Xu^1,2,3^, Ling Ye^1,2,3^, Xuedong Zhou^1,2,3^, Liwei Zheng^1,2^#

1. State Key Laboratory of Oral Diseases, National Clinical Research Center for Oral Diseases, Department of Pediatric Dentistry, West China Hospital of Stomatology, Sichuan University, Chengdu, China

2. West China School of Stomatology, Sichuan University, Chengdu, China

3. National Key Clinical Specialty on Endodontics, West China Hospital of Stomatology, Sichuan University

# Corresponding author: Liwei Zheng

Zheng Liwei: DDS, PhD, State Key Laboratory of Oral Diseases, National Clinical Research Center for Oral Diseases, Department of Pediatric Dentistry, West China Hospital of Stomatology, Sichuan University, No.14, Section 3, South Renmin Road, Chengdu, China, 610041.

Telephone: 862885503469

Fax: 862885503469

1. mail: [liwei.zheng@scu.edu.cn](mailto:liwei.zheng@scu.edu.cn)

**Supplemental Figure and Figure Legends**

**Supplemental Figure 1**


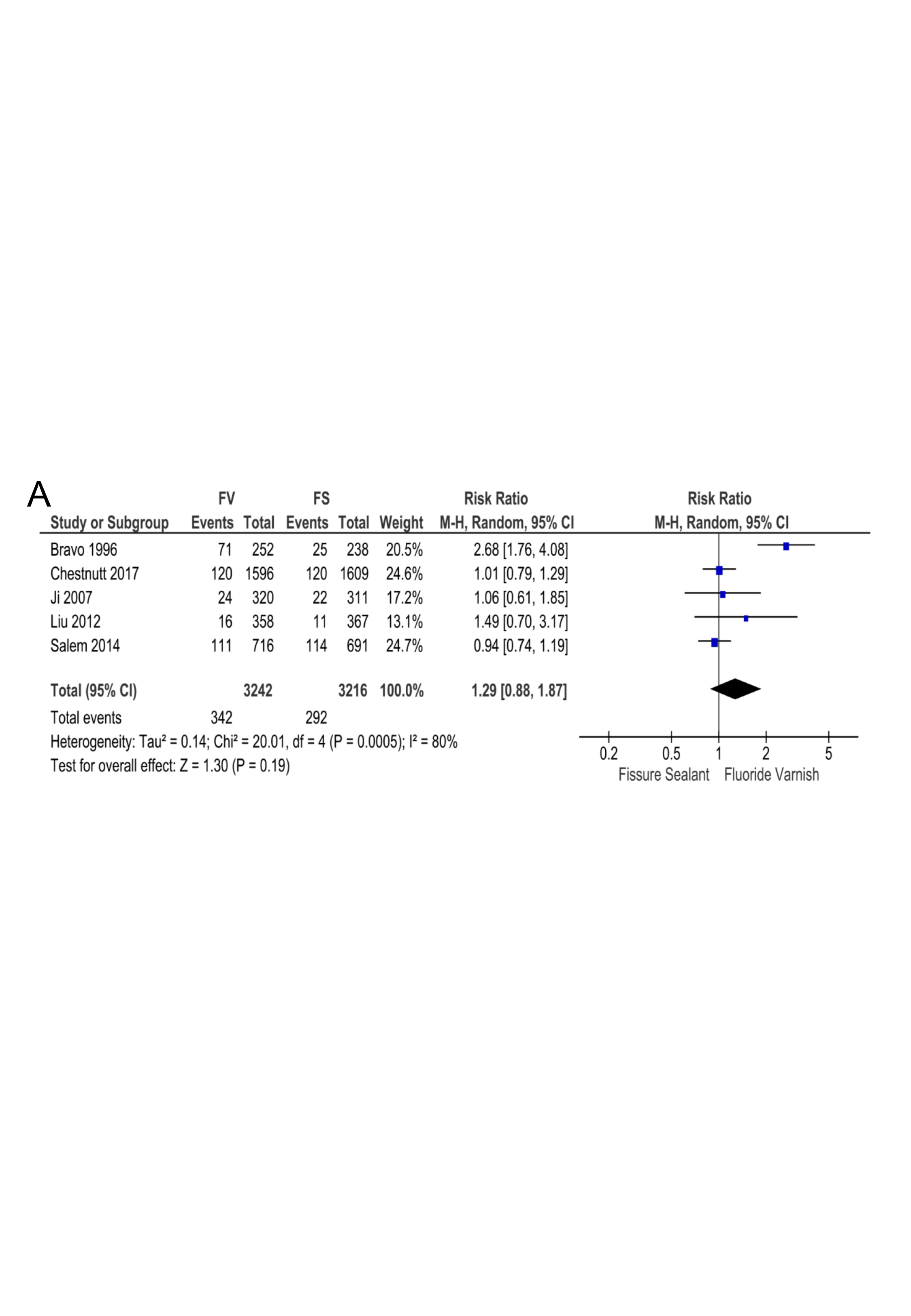


Fig. S1. Subgroup meta-analysis without RCT of split-mouth design. Forest plot of comparison: FS groups was compared with FV groups with respect to CI of FPMs after 2~3 years of follow-up.

**Supplemental Figure 2**


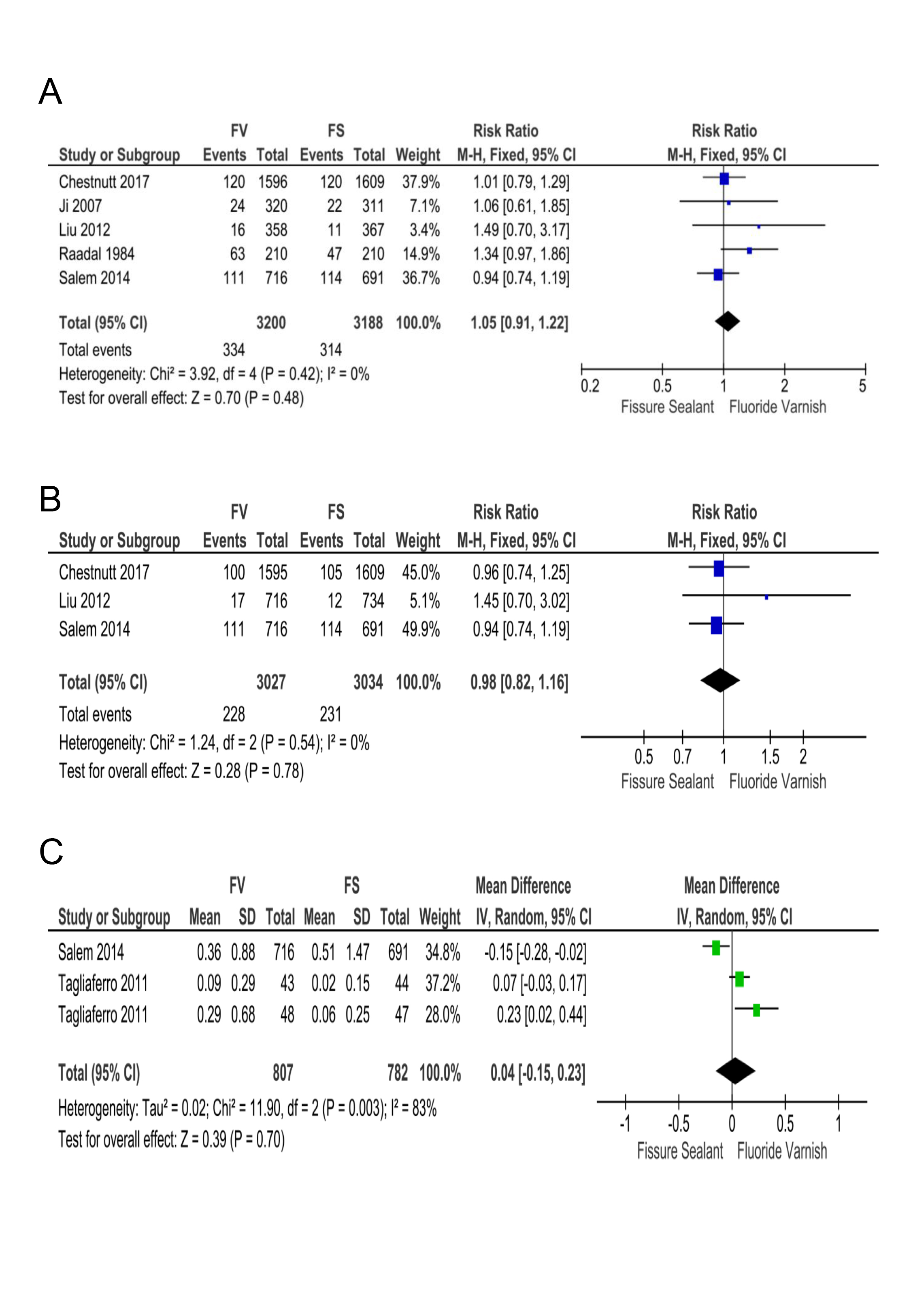


Fig. S2. Subgroup meta-analysis excluded studies from Bravo et al. Forest plot of comparison: FS groups was compared with FV groups with respect to (A) CI of FPMs (B) CI of FPMs’ occlusal surfaces (C) occlusal DMFS increment of FPMs after 2~3 years of follow-up.
